# The Relationship between Physical Activity, Mobile Phone Addiction, and Irrational Procrastination in Chinese College Students

**DOI:** 10.3390/ijerph18105325

**Published:** 2021-05-17

**Authors:** Mengyao Shi, Xiangyu Zhai, Shiyuan Li, Yuqing Shi, Xiang Fan

**Affiliations:** 1 Department of Physical Education, Shanghai Jiao Tong University, Shanghai 200240, China; smy0214@sjtu.edu.cn (M.S.); lishiyuan2019@sjtu.edu.cn (S.L.); stangyuan@sjtu.edu.cn (Y.S.); 2 Graduate School of Sport Sciences, Waseda University, Saitama 359-1192, Japan; xiangyu.zhai@akane.waseda.jp; 3 Shanghai Research Center for Physical Fitness and Health of Children and Adolescents, Shanghai University of Sport, Shanghai 200438, China

**Keywords:** physical activity, mobile phone addiction, procrastination, college students, China

## Abstract

The aim of the current study was to examine the associations between physical activity, mobile phone addiction, and irrational procrastination after adjustment for potential confounding variables. The participants were 6294 first- and second-year students recruited as a cluster sample from three public universities in Shanghai, China. Physical activity, mobile phone use, and irrational procrastination were assessed using the International Physical Activity Questionnaire-Short Form (IPAQ-SF), the mobile phone addiction index scale (MPAI), and the irrational procrastination scale (IPS). The participants were divided into four groups according to their mobile phone usage status and physical activity level. The binary logistic regression model was used to predict the probability of serious irrational procrastination among different groups. The emergence of serious of irrational procrastination under physical activity of different intensity and different mobile phone addiction statuses was predicted by a multiple linear regression model. In this study, the combination of insufficient physical activity and mobile phone addiction is positively associated with high levels of irrational procrastination. Furthermore, students who exhibited both mobile phone addiction behaviors and insufficient physical activity tended to have significantly higher odds of reporting high levels of irrational procrastination than those students who exhibited one behavior or neither behavior. After adjusting for the effects of age, BMI, tobacco, alcohol use, and sedentary time, the result is consistent with previous outcomes. These findings suggest that intervention efforts should focus on the promotion of physical activity and reduction of mobile phone addiction.

## 1. Introduction

Irrational procrastination is defined as voluntary postponement of an intended action even though the individual knows they will be worse off as a result of the postponement [1]. In the late 1970s, irrational procrastination was officially demarcated in the field of psychology, and procrastination behavior was considered an explicit manifestation of some mental health conditions [2]. Previous evidence has shown that serious procrastination can cause serious problems in people’s studies, work, and life, including strong feelings of remorse, guilt, and constant self-denial [3], and lead to increased pressure, depression, anxiety, and fatigue [4]. A British study has indicated that the more urban people procrastinate, the greater their health problems [5]. Procrastination is a common behavior among both teenagers and adults [6]. Globally, people of all ages, especially college students, are negatively impacted by irrational procrastination [7]. While one study reports approximately 70–80% of college students experience various degrees of procrastination [8], another indicates that the prevalence of procrastination among college students is as high as 85.9%, and it is even higher in China [9,10]. Previous studies have shown that the interaction between a series of behavioral, cognitive, and psychological elements may lead to procrastination [11], including addition behavior, stress, anxiety, and depression [12,13].

Healthy lifestyle behaviors are closely related to the physical and mental health of college students. In particular, physical activity has been found to help reduce various mental illnesses [14], and it is a readily available and low-cost treatment. Following the latest 2020 report by the World Health Organization (WHO), increasing physical activity can improve both physical and mental health [15]. However, in 2016, approximately 28% of adults, including college students around the world, were insufficiently physically active [16]. Previous studies have found a correlation between physical activity and procrastination among college students: physical activity has a positive effect on irrational procrastination [9]. However, the effects of different intensities of physical activity have on procrastination have not yet been investigated. Therefore, we used the irrational procrastination scale (IPS) and the International Physical Activity Questionnaire-Short Form (IPAQ-SF) to measure more participants than the previous studies have measured, aiming to find a more accurate relationship between physical activity and irrational procrastination.

Young people aged 18 to 22 are the largest and fastest growing group of users of mobile phones [17]. Contemporary college students tend to study in a paperless and electronic manner, and mobile phones are widely used in life and study [18]. Normal cell phone use can improve efficiency and have a positive impact on mental health, but the consequences of excessive use of mobile phones prove detrimental to mental health, including but not limited to the overload, sleep disorders, physical problems such as neck pain, role conflict, and being unable to respond to all calls and text messages and the subsequent feeling of guilt [19,20].

Previous evidence has shown that problematic smartphone usage negatively affects procrastination [21,22]. Besides, female college students exhibited more serious mobile phone addiction than male college students [23]. However, few existing studies have discussed the impact of physical activity and mobile phone addiction on irrational procrastination among college students. Since engaging in physical activity and using mobile phones have become part of people’s daily routines, considering these behaviors in tandem is necessary in order to develop interventions aimed at reducing procrastination.

The purpose of this study was to investigate the association between physical activity and mobile phone addition combined with irrational procrastination among Chinese college students. We monitored both the physical activity and mobile phone addiction status of our participants. In this paper, we also discussed the effects of different intensities of physical activity on the participants’ procrastination behaviors. Due to factors such as physical activity, mobile phone addiction, and irrational procrastination having different effects on different genders, we specifically discuss men and women separately.

## 2. Participants and Procedure

### 2.1. Participants and Ethical Considerations

This cross-sectional research study was conducted in October 2020. The sample population included all the healthy first- and second-year students enrolled in three universities in Shanghai, China. We distributed 8546 questionnaires and collected 6786 completed questionnaires on a voluntary basis. A total of 492 incomplete questionnaires (7.25% of those collected) were excluded from our sample. Ultimately, a total of 6294 participants with an average age of 18.57 ± 1.82 years were voluntarily recruited in this survey. There were more male participants (*n* = 4,310, 68.48%) than female participants (*n* = 1984, 31.52%), and it included a total of 3463 first-year students and 2831 second-year students.

According to the standardized survey administration protocol, the survey was managed by trained research assistants in laboratories of the participating universities between 11 September and 11 October 2020. The students completed the questionnaire online after having the methods, process benefits, and possible inconveniences explained to them by the research assistants. The participants were provided sufficient time to answer the questions and were instructed how to fill out the survey, including detailed instructions on how to answer the questions. All the students provided informed consent and expressed their willingness to participate. The questionnaire contained data including sociodemographic and anthropometric characteristics, levels of physical activity, mobile phone addiction, and procrastination. Finally, the participants completed a physical fitness test under the supervision of trained testers. This study was approved by the ethics committee of Shanghai Jiao Tong University (NO.H2020043I).

### 2.2. Methods

#### 2.2.1. Sociodemographic and Anthropometric Characteristics

The sociodemographic characteristics of the participants, including gender, age, and grades, were self-reported by the participants. Their anthropometric characteristics, including height and weight, were surveyed after questionnaire completion using an objective measuring instrument (HK6800-ST, Hengkang, Shenzhen, China). BMI was calculated using an internationally accepted method: a person’s weight (kilograms) divided by their height in meters squared (kg/m^2^). The participants were required to be barefoot when measured for height, and height measurements were accurate to 0.1 cm; weight measurements were accurate to 0.1 kg.

#### 2.2.2. Physical Activity

The physical activity of the participants was evaluated by issuing the IPAQ-SF [24,25], which is a reliable and effective physical fitness measurement tool [26]. The participants were required to provide information about the frequency and duration of their exercise, as well as the quality of the activity (vigorous or moderate) and the amount of light physical activity and time spent sitting over the preceding seven days. Among their responses, vigorous-intensity physical activity (VPA) was defined as performing at six or more METs (metabolic equivalents of task). On a scale relative to the individuals’ personal capacities, VPA is usually ranked at 7 or 8 on a scale of 0–10. Moderate intensity physical activity (MPA) refers to physical activity performed between three to six times the intensity of rest. Light-intensity physical activity (LPA) refers to activities with an energy cost of less than three times the energy expenditure at rest for that person, including slow walking, bathing, or other incidental activities that do not result in a substantial increase in heart rate or breathing rate [15]. The different intensities of the participants’ physical activities are analyzed in Section 3 and Section 4.

The data reporting vigorous exercise and moderate exercise exceeding 180 min/day [27] were excluded from this study. According to the latest recommendations of the WHO, adequate physical activity for adults is defined as 300 min of moderate activity, 150 min of vigorous activity, or an equivalent combination of both types of physical activity per week [15]. Therefore, the participants who conformed to the above recommendations were classified under the sufficient physical activity group. Otherwise, they were classified as having insufficient physical activity. 

#### 2.2.3. Mobile Phone Addiction

The mobile phone addiction index scale (MPAI) was used to investigate the participants’ levels of mobile phone addiction [28], which has good reliability and validity among Chinese college students (Cronbach’s α: 0.86) [29]. The MPAI, revised by Leung et al., is a survey regarding mobile phone use with a total of 17 questions. Each question was assigned a score based on a five-point scale. The higher the score, the more addicted the participants are to their mobile phones. In addition, questions 3, 4, 5, 6, 8, 9, 14, and 15 were mobile phone addiction screening questions. If the participants responded to five or more questions with a rating of three or above, they were considered mobile phone addicts. The others were treated as non-phone addicts.

#### 2.2.4. Irrational Procrastination

Procrastination can be conceptualized in terms of general procrastinating traits (measured, for example, by the general procrastination scale and the irrational procrastination scale) and task-specific procrastinating propensity (measured, for example, by the Procrastination Assessment Scale for Students and the bedtime procrastination scale [30,31]. Data regarding the irrational procrastination of the participants were collected using the IPS. This version of the IPS has been verified to have good reliability and validity among Chinese college students [32], and the Cronbach’s alpha for the current study was 0.79. The scale consisted of nine items, each using five-point Likert scoring, wherein “1” and “5” meant “strongly disagree” and “strongly agree”, respectively. Reverse scoring was used for three of the items, and the total score ranged from 0 to 36. The higher the score, the more serious was the participant’s irrational procrastination behavior. This scale has been tested and is applicable to the survey and research of college students in China [32]. Irrational procrastination behavior was divided into serious irrational procrastination (i.e., score > 18), slight irrational procrastination (i.e., 0 < score ≤ 18), and no irrational procrastination (i.e., score = 0) according to the theoretical median number [33].

#### 2.2.5. Lifestyle Behaviors

Smoking and drinking status were determined by the participants’ self-reported responses to the questionnaires’ questions about the participants’ tobacco and alcohol use “Have you ever smoked?” and “Have you ever had alcohol?”. The participants who answered “always” were segmented within the “smoker group” or “drinker group”, and everybody else were segmented within the “no tobacco users” or “no alcohol users”.

### 2.3. Statistical Analyses

The demographic information of the participating students was described using means, standard deviations, and percentages. Furthermore, age, BMI, physical activity time, and MPAI and IPS scores were determined using *t*-tests. The use of tobacco and alcohol, mobile phone addiction conditions, physical activity level, and degree of irrational procrastination between the genders were examined using χ^2^ tests. Normality of the data was checked using the Kolmogorov–Smirnov test; if the skewness coefficient (SC) and the kurtosis coefficient (KC) were each between −1.96 and +1.96, approximate normal distribution could be established [34]. PA_male_, SC = 0.99, KC = 0.63; PA_female_, SC = 1.11, KC = 0.98; MPAI score_male_, SC = −0.03, KC = 0.08; MPAI score_female_, SC = −0.19, KC = 0.06; IPS score_male_, SC = −0.05, KC = 0.15; IPS score_female_, SC = −0.05, KC = −0.01; all of them were normally distributed. Before the *t*-test, homogeneity of variance was checked by the Levene’s test (*p* > 0.05 was variance homogeneity). If the result shows homogeneity of variance, read the *t*-test result in the “Equal variances assumed” row. If the result shows variance non-homogeneity, read the *t*-test result in the “Equal variances not assumed” row. The Pearson correlation test was used to examine the correlation between the time spent engaging in physical activity and irrational procrastination scores and the relationship between MPAIs and IPSs. The binary logistic regression model was used to predict the probability of serious irrational procrastination among different groups. The emergence of serious of irrational procrastination under physical activity of different intensity and different mobile phone addiction statuses was predicted by a multiple linear regression model. Depending on whether physical activity was sufficient or not and the two conditions of mobile phone addiction (addicts and non-addicts), the participants were divided into four different combination groups (i.e., Group I: non-mobile phone addicts + sufficient physical activity; Group II: mobile phone addicts + sufficient physical activity; Group III: non-mobile phone addicts + insufficient physical activity; Group IV: mobile phone addicts + insufficient physical activity). Each combination group (Group I, ref.) was formed after adjusting for age, BMI, smoking and drinking status, and sedentary time per day. The acceptable threshold of statistical significance was specified as *p* < 0.05, and statistical significance was set at *p* < 0.01. The collected data were statistically analyzed using version 25 of the IBM Statistical Package for the Social Sciences (SPSS) software (International Business Machines), and normality and homoscedasticity were also checked by SPSS 25.0 (IBM, Armonk, NY, USA).

## 3. Results

The total of 6294 student participants joined this study. The average BMI of those who conformed to the standard was 22.21 ± 3.61. The participants’ average daily time spent engaging in moderate to vigorous physical activity was 59.48 ± 36.98 min, and 74.2% had sufficient physical activity levels. The physical activity times and levels (rate of sufficiency) of men were significantly higher than those of women (F = 4.51, *p* = 0.034). The average sedentary time of the participants was 486.20 ± 154.46 min, and in this regard, there was no significant difference between male and female students (F = 1.21, *p* = 0.271). The average score of the MPAI was 29.16 ± 12.66 points. According to the mobile phone addiction screening questions, 41.2% of participants were deemed mobile phone addicts, and the degree of mobile phone addiction among male students was significantly lower than that among female students (F = 6,83, *p* = 0.018). The average score of the participants’ IPS was 17.32 ± 5.85 points. Of the college students, 53% displayed serious irrational procrastination behavior, and only 0.2% displayed no procrastination behavior at all. The proportion of serious procrastination behavior among male students was slightly higher than among female students. There was no statistical difference between the IPS scores of male and female students (F = 1.65, *p* = 0.200). Table 1 shows demographic information and other basic statistical information about the participants.

Table 2 showed a significant positive correlation between the participants’ MPAI and IPS scores (*p* < 0.01). The higher the degree of mobile phone addiction, the higher the IPS score. The IPS scores of mobile phone addicts were significantly higher than those of non-mobile phone addicts, and the results showed a significant difference in the IPS scores of mobile phone addicts and non-mobile phone addicts (*p* < 0.01) among both men and women.

**Figure 1:**
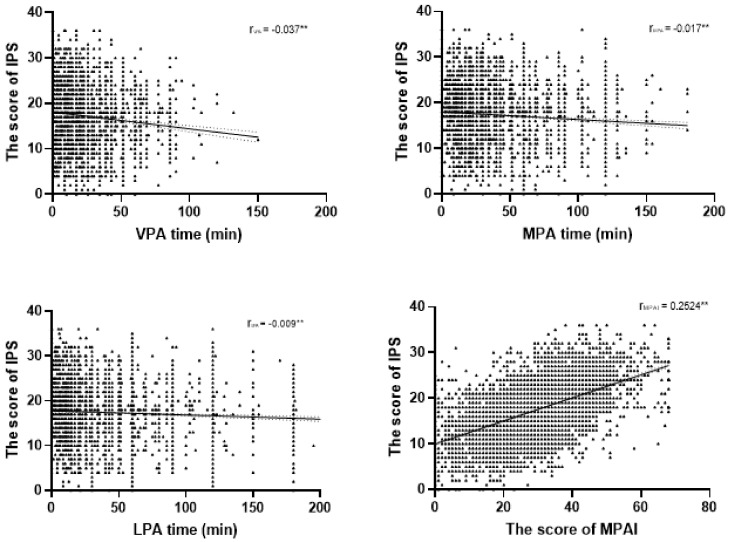
The correlation between the degree of irrational procrastination and the different intensities of physical activity and mobile phone addiction status (** *p* < 0.01).

Furthermore, we contrasted the linear correlations between different intensities of physical activity and irrational procrastination among college students. The duration of VPA was significantly negatively correlated with the participants’ irrational procrastination scores (β = −0.107). The absolute value of r was the highest among all the intensities. Similarly, MPA and LPA were also negatively associated with irrational procrastination (MPV: β = −0.083; LPA: β = −0.069). In Figure 1, a linear correlation slope can be seen for the three intensities of physical activity and irrational procrastination scores. The conclusion to be drawn is as follows: the higher the intensity of physical activity, the greater the degree of its correlation with irrational procrastination. In addition, we found a significant positive correlation between the amount of sedentary time on workdays and irrational procrastination (r = 0.086).

Table 3 shows results of the multiple linear regression analysis which was used to test the predicted relationship between the IPS scores and the different intensities and durations of physical activity and the MPAI score. VPA (β = −0.043; 95% CI = −0.069~−0.017) and MPA (β = −0.033; 95% CI = −0.059~−0.006) were significantly negatively correlated with irrational procrastination in male students, while MPA (β = −0.053; 95% CI = −0.092~−0.014) was negatively associated with the IPS score in both genders, and the MPAI score showed a significant positive correlation with the IPS score for both male and female students (βmale = 0.526; 95% CI = 0.500~0.552; βfemale = 0.554; 95% CI = 0.516~0.592) even after adjusting for age, BMI, tobacco use, alcohol use, and sedentary time. The adjusted scores explained that the variance (R2) of the model for male and female students was 0.299 and 0.333, respectively, and the regression models were statistically significant for both genders.

Table 4 presents the binary logistic regression models, which show the odds of serious irrational procrastination under different combinations of physical activity conditions and mobile phone addiction statuses. All the students were classified into four groups according to their mobile phone addiction statuses and physical activity levels. Group I was specified as the reference group (non-mobile phone addicts × sufficient physical activity). Studies have shown that lack of physical activity or being addicted to the phone increases the odds of serious irrational procrastination. When we further controlled for the effects of age, BMI, tobacco use, alcohol use, and sedentary time, male students with excessive mobile phone usage and insufficient physical activity respectively saw 3.25 (95% CI = 2.793~3.771) times and 1.35 (95% CI = 1.094~1.659) times increased odds of reporting serious irrational procrastination. Compared to the control groups, serious irrational procrastination in men with both mobile phone addiction and insufficient physical activity was 4.26 (95% CI = 3.418~5.312) times higher.

Similar to male students, insufficient physical activity and mobile phone addiction also had a significant positive correlation with serious irrational procrastination among female students even after adjusting for age, BMI, tobacco use, alcohol use, and sedentary time. The presence of both insufficient physical activity and mobile phone addiction increased the odds of serious irrational procrastination by 4.18 times (95% CI = 3.105~5.555) compared with the reference group. Female students with mobile phone addiction only had 3.22 (95% CI = 2.537~4.094) times higher odds of serious irrational procrastination as compared to non-mobile phone addicts and those who engaged in sufficient physical activity. However, among the female students who were not mobile phone addicts, insufficient physical activity was not significantly correlated with serious irrational procrastination when compared to those who engaged in sufficient physical activity.

## 4. Discussion

The purpose of this study was to investigate the prevalence and correlation between insufficient physical activity and mobile phone addiction, as well as the presence of irrational procrastination among Chinese college students. In this study, the IPS scores of the participants were 17.32 ± 5.85, and only 0.2% of the participants exhibited no irrational procrastination. This is consistent with the assertions of previous studies which stated that the occurrence rate of procrastination among college students reached over 95% [10]. In addition, we found that the prevalence of serious irrational procrastination (53.0%) was similar to that reported in previous studies on college students (over 50%) [4] and that the odds of irrational procrastination in this group were higher than those among teenagers (over 40%) [35]. We believe that those variances have to do with the educational stage and lifestyles of the students who participated in the study. All the participants in the present study were first- or second-year students from universities in China; they had just left high school where their learning was passive and moved into new independent lives and learning styles. Numerous changes in living conditions, various academic pressures, and being away from parents or others who could supervise them, they had to face many issues themselves and deal with their emotional burden, which was all completely different from their previous experience. In view of this, procrastination could become an ineffective coping strategy to counteract their problems. This may have led to an increased procrastination behavior among them. The incidence of insufficient physical activity in our investigation (25.8%) is also higher than the national Chinese average (14.1%) [16]; heavy college workload could be a potential reason for this finding as well. The revised WHO guidelines on physical activity and sedentary behavior may also have led to these differences.

The MPAI scores of the study participants were 29.16 ± 12.66. According to the MPAI’s mobile phone addiction screening questions, 41.2% were considered mobile phone addicts; this finding is significantly higher than those of previous studies conducted in China [36] (21.3%) and other countries [37]. The reason for this could be that the increasing daily functions of mobile phones mean that people use them more frequently and for longer periods. Mobile phones are widely used around the world. However, compared with traditional mobile phones, smartphones with their numerous functions and multiple modes of use are superior [38,39]. Today, we are all immersed in a digital world of new technologies that are also making their way into the education process. The root causes of procrastination are most likely related to this deeper change in civilization and educational systems.

Students today are born into the digital world, and they learn how to operate it at an early age. At school, they enter an environment completely different from the family setting: there are many children of the same age who they need to communicate with in college. Using a mobile phone may also be the easiest way for them to seek more social interaction. Contemporary college students tend to study in a paperless and electronic manner [40] and as a result, their screen time and rates of mobile phone addition are also increasing [19]. This view could explain the findings of this study—that there exists a significant correlation between mobile phone addiction and irrational procrastination, thus tracing a serious increase in irrational procrastination. At the same time, the appearance of irrational procrastination behaviors is an explicit manifestation of some mental health conditions [2]. According to previous studies, mobile phone addiction is likely related to anxiety, stress, and sleep quality [41,42], and such studies have also shown an association between screen time and mental health [43]. Therefore, we posit that mobile phone addiction affects mental activities and mental health, thus causing irrational procrastination; this could be a potential mechanism for the correlation between mobile phone addiction and irrational procrastination.

In the present study, the level of physical activity among the participants was significantly correlated with irrational procrastination. The IPS scores of the participants with sufficient physical activity were significantly lower than those of the participants with insufficient physical activity. Similar to Zhong’s study, we found that physical activity is correlated with procrastination among college students [9]. In addition, we found that the intensities of physical activity that the students engaged in were significantly correlated with the effect of physical activity on irrational procrastination. Figure 1 illustrates that higher intensities of physical activity had a greater effect on irrational procrastination. Previous studies have shown that higher intensities of physical activity result in a greater improvement in suppressing passive emotion; this suppression of passive emotions could be a potential reason for physical activity preventing procrastination [41,44]. Numerous studies have documented the correlation between passive emotion—such as anxiety [45], stress [5], and depression [11,46]—and procrastination.

In addition, more time spent engaging in VPA and MPA significantly contributed to lower degrees of irrational procrastination among male students. In contrast, among female students, more time spent engaging in MPA was seen to significantly contribute to a lower degree of irrational procrastination, but the fitting effect of VPA on irrational procrastination was not significant. Studies have found that female students prefer moderate physical activity, such as dance or gymnastics, as their daily exercise [47]. This greater inclination toward moderate physical activity could be a potential reason for MPA being the best exercise intensity to minimize irrational procrastination.

However, after controlling for mobile phone addiction, we found that physical activity levels were significantly correlated with serious irrational procrastination among male but not female students. We also found previous evidence stating that students of different genders have different physical activity habits, which could be one of the main reasons for our finding [47,48]. In particular, even though we found no link between physical activity levels and serious irrational procrastination in female students after controlling for mobile phone addiction, the participants who were both insufficiently active and mobile phone addicts also reported more serious irrational procrastination than those with only a mobile phone addiction. It is possible that these participants took longer to engage in physical activity and instead used their mobile phones, which increased their irrational procrastination behavior. In other words, our study suggests that females who engage in insufficient physical activity could indirectly increase their odds of experiencing serious irrational procrastination.

Furthermore, this study determined that a relationship exists between insufficient physical activity, mobile phone addiction, and irrational procrastination, even after adjusting for the effects of age, BMI, tobacco use, alcohol use, and sedentary time. The presence of both insufficient physical activity and mobile phone addiction significantly increased the odds of irrational procrastination when compared with the presence of behaviors from other categories. Meanwhile, mobile phone addiction had a significant positive correlation with serious irrational procrastination even after controlling for the adjustment factor and physical activity time.

## 5. Conclusions

In this study, irrational procrastination was found to display a significant correlation between both physical activity and mobile phone addiction. Therefore, from a public health and behavior perspective, if college students want to reduce irrational procrastination to improve efficiency, increasing physical activity and reducing mobile phone addiction are vital steps.

The main strength of the present study is that it considers the effects of different intensities of physical activity on irrational procrastination; physical activity and mobile phone addiction are included as mutually confounding factors and are seen as a whole; we also discuss their impact on irrational procrastination separately by studying a large sample of Chinese college students.

One limitation of our present study is the cross-sectional research design: the possibility of reversal causation cannot be ruled out. Another limitation involves the self-reported measures; although they were assessed using standardized questionnaires, this method of information collection is susceptible to measurement errors, memory bias, and the social expectation effect. Therefore, future research should use more objective methods of data collection and measurement, such as accelerometers and mobile monitoring software. Another limitation is that the classification of covariates such as smoking and drinking was not clear enough and should be considered more comprehensively in future research.

## Figures and Tables

**Table 1:** Participants’ demographics and characteristics (*n* = 6294).

Participant Characteristics		Total (*n* = 6294)	Men (*n* = 4310)	Women (*n* = 1984)	*p*-Value
		*n* (%) or Mean ± SD			
Age (years)		18.57 ± 1.82	18.60 ± 1.84	18.50 ± 1.75	<0.01 *
BMI (kg/m^2^)		22.21 ± 3.61	22.83 ± 3.77	20.89 ± 2.81	<0.01 *
Physical activity					
	Physical activity time	59.48 ± 36.98	61.31 ± 37.37	55.51 ± 35.79	<0.01 *
	Sufficient	4668 (74.2)	3328 (77.2)	1340 (67.5)	<0.01 ^†^
	Insufficient	1626 (25.8)	982 (22.8)	644 (32.5)	
Sedentary time		486.20 ± 154.46	486.23 ± 157.53	486.14 ± 147.62	<0.01 *
Mobile phone addiction					
	MPAI score	29.16 ± 12.66	28.47 ± 12.81	30.66 ± 12.20	<0.01 *
	Addicts	2591 (41.2)	1721 (39.9)	870 (43.9)	<0.01 ^†^
	Non-addicts	37035 (8.8)	2589 (60.1)	1114 (56.1)	
Irrational procrastination					
	IPS score	17.32 ± 5.85	17.35 ± 5.82	17.26 ± 5.89	<0.01 *
	Serious procrastination	3336 (53.0)	2328 (54.0)	1008 (50.8)	<0.01 ^†^
	Slight procrastination	2944 (46.8)	1974 (45.8)	970 (48.9)	
	No procrastination	14 (0.2)	8 (0.2)	6 (0.3)	
Tobacco use					<0.01 ^†^
	Never	6208 (98.6)	4241 (98.4)	1967 (99.1)	
	Yes	86 (1.4)	69 (1.6)	17 (0.9)	
Alcohol use					<0.01 ^†^
	Never	4298 (68.3)	2755 (63.9)	1543 (77.8)	
	Yes	1996 (31.0)	1555 (36.1)	441 (22.2)	

Note: *p* *, *t*-tests; *p* ^†^, χ^2^ test; BMI, body mass index; MPAI, mobile phone addiction index scale; IPS, irrational procrastination scale. All values represent raw non-standardized scores.

**Table 2:** The Pearson correlation coefficient for the degree of irrational procrastination exhibited by the participants (IPS score) and across different groups.

Participant Characteristics		Total (*n* = 6294)	Men (*n* = 4310)	Women (*n* = 1984)
		Mean ± SD		
Physical activity				
	Sufficient	16.97 ± 5.87 **	17.05 ± 5.86 **	16.76 ± 5.90 **
	Insufficient	18.34 ± 5.64 **	18.36 ± 5.57 **	18.32 ± 5.74 **
Mobile phone addiction				
	Addicts	19.97 ± 5.10 **	20.04 ± 5.06 **	19.83 ± 5.19 **
	Non-addicts	15.47 ± 5.61 **	15.56 ± 5.61 **	15.26 ± 5.61 **

Note: **, *p* < 0.01; *p*-value for significant IPS score differences between the different groups determined using a *t*-test.

**Table 3:** The Pearson correlation coefficient for the degree of irrational procrastination exhibited by participants (IPS score) and across different groups.

Variable	Male (Adjusted R^2^ = 0.299 **)		Female (Adjusted R^2^ = 0.333 **)	
	β (95% CI)	*p*-Value	β (95% CI)	*p*-Value
LPA	−0.026 (−0.052~−0.001)	0.057	−0.014 (−0.053~0.025)	0.491
MPA	−0.033 (−0.059~−0.006)	0.017	−0.053 (−0.092~−0.014)	0.007
VPA	−0.043 (−0.069~−0.017)	0.001	−0.034 (−0.073~0.004)	0.080
MPAI	0.526 (0.500~0.552)	0.000	0.554 (0.516~0.592)	0.000

Note: adjusted for age, BMI, tobacco use, alcohol use, and sedentary time. ** *p* < 0.01.

**Table 4:** The odds of serious irrational procrastination under different combinations of physical activity conditions and mobile phone addiction statuses.

Group	Men	Women		
	OR (95% CI)	aOR^a^ (95% CI)	OR (95% CI)	aOR ^a^ (95% CI)
Group I	1 (ref.)	1 (ref.)	1 (ref.)	1 (ref.)
Group II	3.325 ** (2.870~3.853)	3.246 ** (2.793~3.771)	3.113 ** (2.469~3.853)	3.223 ** (2.537~4.094)
Group III	1.350 ** (1.101~1.655)	1.347 ** (1.094~1.659)	1.229 (0.920~1.640)	1.229 (0.967~1.746)
Group IV	4.510 ** (3.632~5.600)	4.261 ** (3.418~5.312)	4.024 ** (3.062~5.288)	4.183 ** (3.105~5.555)

Note: ^a^ adjusted for age, BMI, tobacco use, alcohol use, and sedentary time; ** *p* < 0.01.

## Data Availability

The data in the study are not publicly available in order to protect privacy of the participants.
